# Cilia-Inspired Bionic Tactile E-Skin: Structure, Fabrication and Applications

**DOI:** 10.3390/s25010076

**Published:** 2024-12-26

**Authors:** Jiahe Yu, Muxi Ai, Cairong Liu, Hengchang Bi, Xing Wu, Wu Bin Ying, Zhe Yu

**Affiliations:** 1 In Situ Devices Center, School of Integrated Circuits, East China Normal University, Shanghai 200241, China; 2 School of Electrical Engineering, Korea Advanced Institute of Science and Technology, Daejeon 34141, Republic of Korea

**Keywords:** bionic e-skins, flexible tactile sensors, cilia-inspired microstructures, fabrication methods, intelligent applications

## Abstract

The rapid advancement of tactile electronic skin (E-skin) has highlighted the effectiveness of incorporating bionic, force-sensitive microstructures in order to enhance sensing performance. Among these, cilia-like microstructures with high aspect ratios, whose inspiration is mammalian hair and the lateral line system of fish, have attracted significant attention for their unique ability to enable E-skin to detect weak signals, even in extreme conditions. Herein, this review critically examines recent progress in the development of cilia-inspired bionic tactile E-skin, with a focus on columnar, conical and filiform microstructures, as well as their fabrication strategies, including template-based and template-free methods. The relationship between sensing performance and fabrication approaches is thoroughly analyzed, offering a framework for optimizing sensitivity and resilience. We also explore the applications of these systems across various fields, such as medical diagnostics, motion detection, human–machine interfaces, dexterous robotics, near-field communication, and perceptual decoupling systems. Finally, we provide insights into the pathways toward industrializing cilia-inspired bionic tactile E-skin, aiming to drive innovation and unlock the technology’s potential for future applications.

## 1. Introduction

Touch is one of the five fundamental senses of human beings, along with hearing, vision, smell, and taste. Touch begins functioning in the unborn fetus and remains actively engaged throughout life, even as the human body ages. Touch allows humans to rapidly perceive rich and multidimensional information about contacted objects, including weight, shape, texture, characteristics, movement trajectory, etc., laying the foundation for assessing environmental conditions and making adaptive adjustments. The skin, as the main medium of touch and the largest organ of the human body, has garnered significant attention from researchers aiming to develop high-performance flexible tactile sensors. These researchers seek to simulate its sensing functions and mechanical properties (softness and elasticity). This innovative flexible tactile sensor, referred to as electronic skin (E-skin) [1,2,3], achieves the accurate capture and efficient processing of pressure signals with the advancement of physical science, chemical science, micro/nano-electronic engineering, and bionic technology, thus showcasing immense potential across various applications, such as embodied artificial intelligence robotics [4,5], neuroprosthetic systems, and real-time health monitoring [6].

Currently, the sensing mechanisms of tactile E-skin [7] are primarily categorized into five distinct types: (1) Resistive sensors [8] convert pressure changes into resistance or current variations, making them the earliest and simplest form of pressure sensors. They are characterized by high sensitivity, stable data output, and ease of integration. (2) Capacitive sensors [9] resemble plate capacitors, comprising an electrode layer and a dielectric layer. When subjected to pressure, the dielectric layer compresses, altering the spacing between the electrodes and increasing the capacitance of the sensor. These sensors boast a wide detection range, seamless integration capabilities, and a diverse range of material options. (3) Iontronic sensors are a new type of sensor used to detect the capacitance of devices, exhibiting sensitivity far beyond that of conventional capacitive sensors because the ionic liquid introduced inside the devices can produce a double-layer effect under pressure. It is more important that this type sensor is free from the interference of capacitive coupling. (4) Piezoelectric sensors convert pressure signals into voltage signals, leveraging piezoelectric materials that, under external force, separate internal positive and negative charges to generate a polarized piezoelectric effect. This enables the detection of external dynamic forces and is characterized by fast response times, high sensitivity, and self-powering capabilities [10,11]. (5) Triboelectric sensors, which are relatively new types of passive pressure sensors introduced in the present century, harness the triboelectric effect. This occurs when different materials come into contact and experience friction, inducing an electric charge on their surfaces, which creates a potential difference. This enables the detection of dynamic forces and these sensors are known for their rapid response, diverse material options, and self-powering capabilities.

E-skin possesses several key performance indicators crucial to its functionality. These include sensitivity, detection limit, hysteresis coefficient, response/recovery time, cycle durability, and dynamic stability [12]. Sensitivity, denoted as S, quantifies the sensor’s ability to convert a pressure stimulus into a discernible electrical signal. It is mathematically defined as the ratio of the change in electrical signal (ΔE) to the change in pressure stimulus (ΔP). This metric is crucial in determining how effectively the sensor responds to tactile inputs. The detection limit refers to the threshold below which the sensor is unable to distinguish a pressure stimulus from background noise or interference. This parameter establishes the minimum pressure required for the sensor to generate a reliable electrical signal. Hysteresis describes the discrepancy in the sensor’s electrical signal response during loading and unloading processes under identical pressure stimuli. It is an important factor influencing the accuracy and repeatability of sensors. Response/recovery time measures the speed at which the sensor’s output signal reaches 90% of its stable value upon rapid pressure stimulation. Given that human tactile response/recovery time is approximately 50 milliseconds, it is generally expected that E-skin sensors meet or exceed this benchmark for seamless integration with human–machine interfaces. Cycle durability assesses the sensor’s ability to maintain its original force-to-electrical response ratio over extended periods of cyclic operation. This parameter is crucial for ensuring the longevity and reliability of E-skin sensors in practical applications. Lastly, dynamic stability refers to the sensor’s capacity to maintain consistent performance under varying conditions and over time, ensuring that it can accurately detect and respond to tactile stimuli in dynamic environments. In response to the demand for above performance enhancement across multiple sensor types, particularly the five primary sensor types mentioned earlier, we summarize a universal method involving the construction of microstructures on the surface of sensitive materials. This method has been proven to effectively improve the performance of related devices, which has emerged as a significant research direction in recent studies.

The design of force-sensitive structures [13] has a decisive impact on device performance. For example, Mannsfeld et al. [14] reported that the maximum slope of the relative capacitance change in the pyramidal film in the 0.2 kPa range is 0.55 kPa^−1^, which is approximately 30 times that of unstructured thin films within the same range. And the structured thin film exhibits millisecond-scale relaxation time upon full-pressure release, whereas unstructured films take over 10 s to revert to their original unloaded state. Furthermore, the microstructured thin film is capable of sensing minute pressures as low as 3 Pa. Microstructures can reduce the material’s inherent viscoelastic characteristics by focusing stress, leading to improved performance. Researchers have embraced the strategy of designing and incorporating a wide array of microstructures onto material surfaces. Examples of such structures include pyramids [15], domes [16,17], and porous configurations [15], each tailored to optimize specific performance metrics. Among these natural wonders, cilia stand out as a microstructure present on the surfaces of cells and organisms. Renowned as “biological sensing antennas”, cilia play a pivotal role in sensing external stimuli [18] owing to their exquisite morphology and intricate arrangement. These features enable them to effectively capture and transmit mechanical signals, empowering organisms to swiftly adapt to environmental variations. The potential of bionic designs inspired by cilia is profound, particularly in the domain of artificial sensors. By replicating cilia-like microstructures [19] in sensors, researchers have achieved remarkable improvements in parameters such as sensitivity and response speed. This bionic approach not only accurately mirrors nature’s force-sensitive mechanisms but also facilitates more precise detection and analysis in intricate environmental conditions. Collectively, the introduction of microstructures and the bionic design of cilia represent a cornerstone in advancing sensor technology. These advancements hold immense promise for applications spanning medicine, environmental monitoring, and smart devices, and may usher in a new era of innovative and responsive sensing solutions.

**Figure 1:**
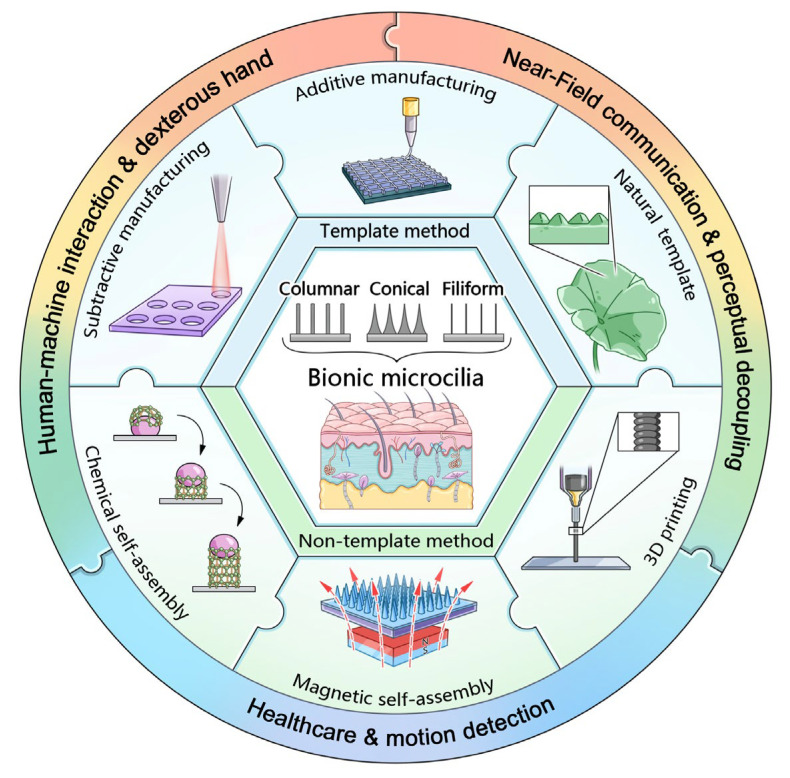
**Overview of cilia-inspired bionic tactile E-skin, illustrating structures, fabrication methods, and applications.** The central image shows bionic microcilia structures, with surrounding sections highlighting template-based and template-free fabrication techniques and applications in human–machine interaction, near-field communication, and healthcare.

In recent years, the field of flexible sensors has witnessed a surge of research focused on exploring various microstructures to enhance sensor performances. However, the unique microstructure of cilia, a biological marvel, has remained relatively understudied despite its immense potential. Recognizing the significance of this biological structure [20,21,22], we endeavor to delve into the definition, classification, preparation methods, and applications of cilia in the context of flexible sensors. Bionic cilia microstructures, with their exceptional properties, have garnered significant attention due to their ability to mimic the sophisticated sensing capabilities of natural cilia. Consequently, this paper aims to consolidate the latest research findings on cilia-inspired E-skin, highlighting the advancements made and the challenges that lie ahead for the next generation of these devices (Figure 1). Structured into three distinct parts, we first explore the specific microstructure types of cilia, examining the performance characteristics of different species. This exploration offers insights into how the intricate architecture of cilia contributes to the overall functionality of these sensors. Due to its high aspect ratio, the cilium microstructure differs from other microstructures. Imagining the preparation of cilium-like microstructures is akin to constructing skyscrapers, which require robust support or else they may easily collapse. This poses significant manufacturing challenges. Therefore, in the second part, we place emphasis on the preparation methods for cilia, critically assessing the merits and drawbacks of the various materials, processes, and technologies employed in their fabrication, and highlight the difficulties in fabricating such structures. This analysis sheds light on the ongoing efforts to optimize the production of bionic cilia microstructures, paving the way for more efficient and cost-effective manufacturing methods. Lastly, we examine the current applications and future development trends in cilia-inspired bionic tactile E-skin across diverse fields, including medical health, motion detection, human–machine interaction and near-field communication. We discuss the practical challenges encountered during implementation and propose potential solutions. Looking ahead, we envision the promising directions for the continued evolution of cilia-inspired bionic tactile E-skin, aiming to inspire new ideas and strategies for the advancement of flexible electronic devices.

## 2. Structure of Artificial Cilia in Bionic Tactile E-Skin

**Figure 2:**
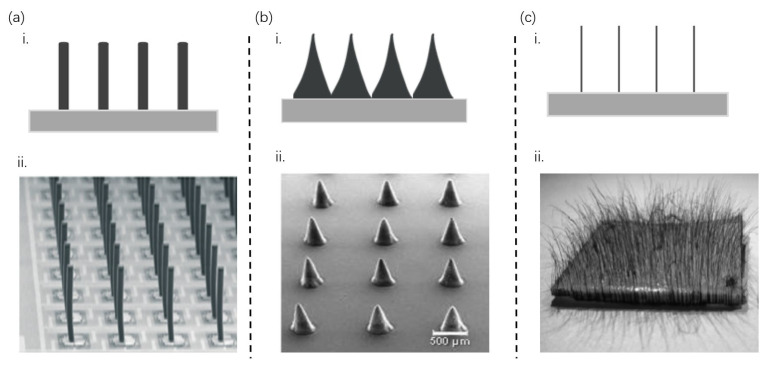
**Structural design of artificial cilia in bionic E-skin.** (**a**) (**i**): Columnar cilia. (**ii**): Array of spiral-suspended sensory hairs, inspired by natural arrangements, to enhance the sensitivity and responsiveness of E-skin. (**b**) (**i**): Conical cilia. (**ii**): SEM images of PDMS films showing the microstructure of conical cilia. (**c**) (**i**): Filiform cilia. (**ii**): Photograph of a PTFE substrate with magnetic cilia mimics, fabricated under a magnetic field of 450 mT with a vertical gradient of 0.1 T/m using an electromagnet. Figure 2a-ii, Figure 2b-ii, and Figure 2c-ii are from ref [23], ref [24], and ref [1], respectively.

In the field of tactile E-skin, bionic artificial cilia have emerged as innovative microstructural designs [13]. They have progressively demonstrated their unparalleled advantages. These artificial cilia are meticulously crafted based on a deep understanding of natural biological cilia, such as those found in organisms like paramecia. With unique morphologies, materials, and functions, these bio-cilia enable exquisite tactile perception in living organisms. Inspired by this natural phenomenon, researchers have skillfully transformed these biological properties into artificial cilia, integrating them into flexible tactile sensors. By carefully replicating the intricate structures and functionalities of biological cilia, these artificial cilia provide the sensors with exceptional sensitivity, remarkable adaptability, and diverse tactile perception capabilities. As a result, these sensors are capable of detecting a variety of physical parameters, including pressure, temperature, humidity, and so on. This chapter explores the microstructure of bionic artificial cilia used in cilia-inspired tactile E-skins. Specifically, we categorize the shapes of cilia into three distinct types—columnar [23], conical [24], and filiform [1] (Figure 2)—discussing their respective applications in tactile perception.

### 2.1. Columnar Cilia

The columnar cilia, with their upright morphology and robust structure, offer exceptional support and directional guidance for the sensor. This type of cilia typically has a substantial cross-sectional area and high rigidity, enabling it to withstand external disturbances to a considerable extent while maintaining the stability and precision of the sensor [25]. In the realm of flexible sensors, columnar cilia frequently serve as a crucial mechanical sensing component, adept at accurately detecting physical quantities [26,27,28,29] such as pressure and vibration by gauging deformation or displacement under applied forces.

**Figure 3:**
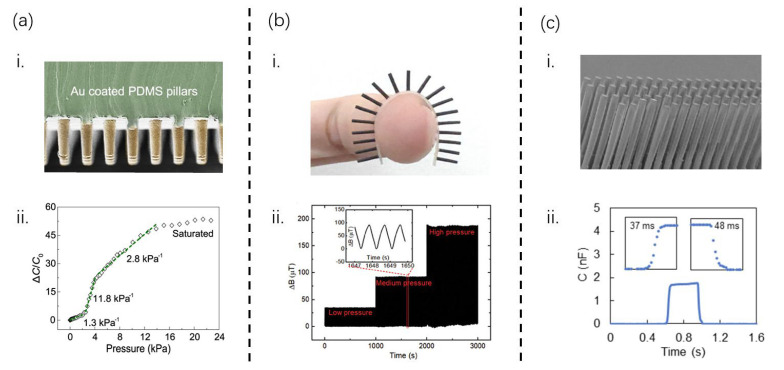
**Columnar cilia structures and their sensing performance**. (**a**) (**i**): An SEM image of the micro-columnar structure. Scale bar: 50 μm. (**ii**): Normalized capacitance change as a function of applied pressure for a skin-electrode mechanosensing structure (SEMS) with a spacer, demonstrating sensitivity to various pressures. (**b**) (**i**): Single-row magnetic cilia in the height of 5 mm. (**ii**): Sensor output during repeated bending under low, medium and high pressures, with the inset showing consistent waveform for three bends, emphasizing reliability. (**c**) (**i**): An SEM image of a prior artificial hair flow sensor array design, with multiple hairs aligned parallelly, sharing a substrate electrode. (**ii**): The sensor response to a 100 g weight, with insets displaying response and recovery times. Figure 3a, Figure 3b, Figure 3c-i, and Figure 3c-ii are from ref [6], ref [30], ref [31], and ref [32], respectively.

Electrodermal devices that capture physiological responses from the skin are essential for monitoring vital signs. However, these devices often require intricate layered designs that incorporate electronically or ionically active materials, relying on elaborate synthesis, encapsulation, and packaging methodologies. Zhu et al. [6] demonstrated that ion transport in living systems can facilitate a streamlined approach to ion-electron sensing, circumventing the need for synthetic ionic materials. This not only simplifies device architecture but also reduces production costs and environmental impact. They devised a simple structure of skin electrode mechanical sensing (SEMS), akin to a dual-capacitor (CE and SE) and resistor system (Figure 3a-i), in which the SE, primarily used for sensing, consists of gold-plated polydimethylsiloxane (PDMS) micro-columnar Cilia. These pillars facilitate a sensitive alteration in the skin-electrode interface’s contact area during loading, thereby modulating capacitance. Notably, despite its minimal initial contact surface and low initial capacitance, the SEMS exhibits remarkable sensitivity. Testing revealed a nonlinear capacitance–pressure response (Figure 3a-ii) spanning four stages, with sensitivity peaking at 1.3 kPa^−1^ below 3 kPa, rising to 11.8 kPa^−1^ between 3 and 4 kPa, tapering to 2.8 kPa^−1^ from 4 to 15 kPa, and ultimately reaching saturation above 15 kPa. Furthermore, it boasts high pressure and high spatial resolutions, enabling the detection of subtle physiological signals such as fingertip pulses under varying skin degrees of humidity. The ability to distinguish between motion-related and pulse signal frequencies effectively reduces motion artifacts in physiological signal monitoring, a critical advancement in terms of achieving wearable devices capable of functioning stably in dynamic environments.

Piezo-resistive tactile sensors are prone to temperature variations, leading to significant device drift. Conversely, piezoelectric tactile sensors require dynamic inputs, rendering them unsuitable for predominantly static haptic forces. Capacitive tactile sensors, while popular, are susceptible to stray capacitance and exhibit unstable outputs over periods of prolonged usage. To address these challenges, Man et al. [30] introduced a magnetic hair array comprising three layers (Figure 3b-i): upper (magnetic cilia), middle (flexible PDMS film), and lower (serpentine FPC with magnetic sensor array). The number and area of magnetic cilia can be tailored for specific applications. Upon force-induced bending, the magnetic sensor array detects changes in the magnetic field, quantifying the magnitude and direction of the external force. This sensor has a 0.2 mN resolution, an operating range from 0 mN to 19.5 mN, and directional force discrimination capabilities. Its expansive sensing area and quick response time make it ideal for glide tactile detection. Stability and dynamic response are essential for flexible sensors. Repeated bending tests (3000 cycles at 1 Hz, under varying forces) confirmed the sensor’s stability under medium to low forces, with minimal drift, even under high bending forces (Figure 3b-ii.).

Dagamseh et al. [31] designed an innovative “flow camera” utilizing an array of bionic cilia flow sensors. By capturing signals from individual sensor units, this system generates a spatiotemporal image of the airflow across the entire hair sensor array. The study investigates various interface scenarios suitable for integrating a single element within the array configuration. Notably, the use of frequency division multiplexing (FDM) for capacitive detection in hair sensor arrays (as depicted in Figure 3c-i) has proven advantageous. Unlike time-division multiplexing (TDM), FDM is immune to propagation delays and synchronization errors, ensuring more reliable and efficient data transmission. Furthermore, FDM offers scalability to larger array structures, maintaining both the acquisition speed and the signal-to-noise ratio (SNR) of individual sensors at optimal levels. This enables the effective interrogation of the capacitive array sensors. Leveraging the advanced silicon-on-insulator technology, coupled with a deep trench isolation structure, the researchers developed a differential capacitive readout hair flow sensor, which was seamlessly integrated into a single-chip microcomputer array. This advancement highlights the potential of this technology for flow sensing applications.

Furthermore, Pan et al. [32] synthesized PVA-H_3_PO_4_ ionic hydrogel films with a columnar and tunable microstructure through an enhanced lithography process. This flexible sensor is notable for its ability to monitor pulse waves without external pressure or cuff compression, offering a non-invasive alternative. It can detect distinct arterial pulse waves and heart rates without applying any pressure and can even monitor pulse waves under varying static pressures, facilitating accurate blood pressure measurements. Notably, the ionic hydrogels used in this sensor are eco-friendly and can be recycled. The hair-like, inherently unstable microstructure of the sensor exhibits remarkable sensitivity, achieving values of 2296 kPa^−1^, 1167 kPa^−1^, and 511 kPa^−1^ within the ranges of 0–4 kPa, 4–40 kPa, and 40–100 kPa, respectively. Particularly in the 4–40 kPa range, it demonstrates both high sensitivity and linearity, making it ideally suited for cuffless blood pressure measurements. These labile microstructures are precisely crafted through photolithography, ensuring consistent quality and high synthesis efficiency. Additionally, the sensor has a low detection threshold of approximately 3.9 Pa and exceptional durability, enduring over 2900 load/unload cycles. To evaluate the response time of this flexible sensor, a test was conducted by applying a counterweight of approximately 100 g. The sensor’s relative capacitance change surged within 37 milliseconds, surpassing the human skin’s response speed (30–50 ms), and then stabilized at a consistent value. Upon removing the counterweight, the sensor promptly returned to its initial state within approximately 48 milliseconds (Figure 3c-ii). This rapid and reliable response highlights the sensor’s potential for real-time monitoring applications.

### 2.2. Conical Cilia

In nature, hairs and cilia predominantly exhibit conical shapes rather than columnar ones [33], granting them exceptional sensitivity in perceiving external pressure. They can utilize their slender tips to amplify and transmit external mechanical stimuli. Scientists have pioneered the development of tapered cilia, characterized by slender tips and broader bases, which significantly amplify deformation or electrical signal changes in response to external stimuli. This inherent attribute of conical cilia underscores their immense potential for widespread adoption in tactile sensors and airflow sensors. Though meticulously fine-tuning of the geometric parameters and material composition of these tapered cilia, it becomes feasible to further elevate the sensitivity of the sensor and shorten its response speed, enabling the precise detection of even the slightest physical variations.

**Figure 4:**
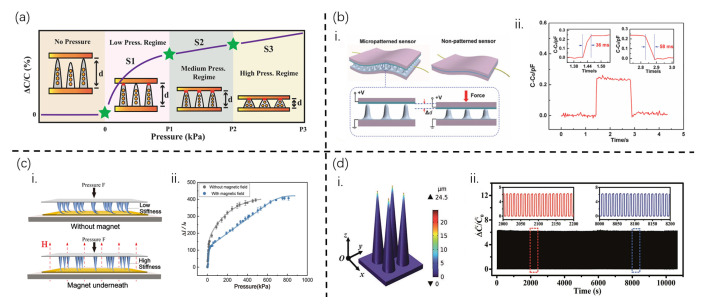
**Conical cilia structures and their sensing performance.** (**a**) The capacitance change mechanism of the sensor with conical cilia microstructure in response to applied pressure, demonstrating its precision and sensitivity in detecting pressure variations. (**b**) (**i**): Schematics showing configuration and sensing mechanisms of both microstructured and non-patterned devices, with the microstructured sensor enhancing sensitivity to tactile stimuli. (**ii**): The real-time response of the sensor to an ultra-low pressure of 0.8 Pa. The insets show the response time upon loading and unloading. (**c**) (**i**): The mechanism of the in situ tuning of the sensor performance. (**ii**): The performance comparison of pressure sensors before and after magnetic field application. (**d**) (**i**): The simulation of stress distribution and the displacement of the MCAs and MPA with vertical pressure. (**ii**): The long-term stability of the pressure sensor under cyclic loading/unloading processes with 200 kPa at a frequency of 5 s, with insets showing signals from 2000–2200 to 8000–8200 s. Figure 4a, Figure 4b, Figure 4c, and Figure 4d are from ref [9], ref [34], ref [8], and ref [35], respectively.

The conical structured cilia, with their unique tapered geometries and gradual geometric gradient, exhibit sensitive responsiveness and gradient perception capabilities to changes in physical quantities. Asghar et al. [9] utilized the self-assembly of a curable magnetorheological fluid (CMRF) film under the influence of a vertically cured magnetic field to create magnetically grown conical cilia. By optimizing the fabrication parameters of the cilia, namely the concentration of magnetic particles (MPs) and magnetic field strength, they achieved a piezoelectric capacitive sensor with an ultra-fast response time (50 ms), high cyclic stability (>9000 cycles), and an extremely low detection limit (1.9 Pa) for a wide range of pressures(from 0 to 145 kPa). The gradual decrease in the cross-sectional area of the conical cilia from the bottom to the top directly results in a trend where the sensitivity of the cilia is initially very high under low applied pressures and gradually decreases thereafter. In the low-pressure region, due to the presence of tiny air gaps and the inconsistent heights of the cilia, the tips of some larger-sized cilia first come into contact with the inner surface of the upper electrode, causing the sensor to exhibit maximum sensitivity (low-pressure region, Figure 4a), as illustrated in low-pressure region. In the medium-pressure region, in addition to the compression of the larger cilia, the tips of the remaining shorter cilia also begin to make contact with the electrode, as depicted in medium-pressure region. At this point, more cilia are in contact with the electrode, and the contact area per micro needle also increases, leading to an overall increase in contact area and consequently lower sensitivity in the medium-pressure region compared to the low-pressure region. Furthermore, during compression, the conical structure undergoes uneven stress distribution, with stress concentrating at its tip, as shown in the high-pressure region. As a result, the tip of the conical structure compresses more due to its smaller area, resulting in greater mechanical deformation at the top. Moreover, the conical structure initially exhibits high sensitivity due to its small contact area, which decreases as the contact area increases under higher compressive pressures, causing the structure to revert to a more truncated state with a larger cross-sectional area. Finally, under high pressure, the cone is fully compressed, and further increases in pressure only lead to minor changes in the contact area, thereby causing minute changes in capacitance with a sensitivity of S3, which is lower than S2. Additionally, high compression may slightly alter the fixed positions of MPs within the micro-needles, affecting the sensor characteristics, resulting in a relatively low detection upper limit for such conical cilia.

Inspired by the hair-like skin of humans, Wan et al. [34] have developed a highly sensitive flexible tactile sensor utilizing lotus leaf-mimicking microstructured PDMS (m-PDMS). The m-PDMS substrate is composed of high-aspect-ratio, low-density microtowers, which are essentially the conical cilia depicted in Figure 4b-i. These cilia exhibit a high sensitivity of 1.2 kPa^−1^, an ultra-low detection limit of 0.8 Pa, and a rapid response time of 36 ms. Finite element analysis reveals that the sparse distribution and high aspect ratio of the conical cilia are crucial factors in achieving high sensitivity, low detection limits, and fast responses to external stimuli. This conical cilia-based flexible tactile sensor also demonstrates high robustness, enduring at least 100,000 cycles of testing without showing signs of fatigue. The unique microtower array structure of the lotus leaf surface, along with the extensive fine hairs on the microtowers, possesses high aspect ratios and low densities, enabling the fabricated conical cilia to mimic this rapid response capability. To investigate the limit of detection (LOD) and response time of the micro-patterned tactile sensor, a thin sheet of paper (20 mg or 0.8 Pa) was uniformly applied across the entire device surface. Upon loading with the lightweight paper, the sensor’s capacitance rapidly increases within 36 ms (Figure 4b-ii), comparable to the response time of human skin (from 30 to 50 ms), before stabilizing. Upon removing the paper, the sensor’s capacitance rapidly decreases, exhibiting a brief recovery component within approximately 58 ms before decaying to its initial value. However, while these cilia respond to minute forces within such short timeframes, it is evident from the graphs that there exists a disparity between the loading and unloading response times, with unloading being slower than loading, which is indicative of hysteresis and a slower rebound in the conical cilia.

Xu et al. [8] have introduced an innovative approach that harnesses the microcavity-induced effect to ultra-rapidly fabricate ordered conical hair arrays under the influence of both static and dynamic magnetic fields. Notably, the stiffness of these conical hairs can be deliberately modulated. Upon the application of pressure (F), the hairs undergo deformation, increasing the contact area between the surface carbon black (CB) layer and the interdigitated electrodes. This, in turn, leads to a reduction in total resistance and an enhancement in output current. Conversely, when a magnetic field is imposed, the hairs’ stiffness increases, resulting in lesser deformation under the same pressure (F), as depicted in Figure 4c-i. Consequently, the output current diminishes compared to its non-magnetic state. To investigate the impact of magnetic field actuation on sensor performance, a CI-MCA (with a length of 800 μm and an average diameter of 200 μm) was employed as the sensing layer, and its performance was evaluated before and after exposure to a magnetic field (200 mT). Sensitivity and linearity were characterized by measuring the change in output current (ΔI/I_0_). In the low-pressure range (0–20 kPa), the sensor exhibited a remarkable sensitivity of 10.2 kPa^−1^, with minimal magnetic influence on this parameter. However, in the medium- to high-pressure region (20–700 kPa), the linearity was significantly improved under magnetic actuation, with the saturation pressure elevated from 450 kPa to 850 kPa, marking a substantial extension of the pressure range. The practical functionality of this stiffness-tunable sensor was verified by applying a 700 g pressure and altering its stiffness with a magnet (Figure 4c-ii). Without a magnetic field, the output current rapidly increased in response to pressure. Upon magnetic field application, the current abruptly decreased, and upon its removal, the original state was restored. This non-contact, in situ adjustment of sensor performance presents a viable solution for achieving its application across diverse scenarios.

Recognizing that triangles are the most stable planar structures, conical hairs, being their three-dimensional rotational counterparts, also possess robust stability. Zhou et al. [35] pioneered hairy microcilia arrays (MCAs) as the dielectric layer to develop a flexible capacitive pressure sensor with high sensitivity and a wide detection range (Figure 4d-i). By manipulating various fabrication parameters, such as magnetic fields and composite material mass ratios, they systematically explored the shape controllability of MCA structures. Upon the optimization of the conical hair design, the sensor achieved an impressive sensitivity of 0.28 kPa^−1^ within 0 kPa and 10 kPa, alongside a broad detection range of up to 200 kPa with a sensitivity of 0.02 kPa^−1^, and a low detection limit of 2 Pa. Additionally, it exhibited exceptional structural robustness and stability. Under a constant pressure of 200 kPa (Figure 4d-ii), the sensor was subjected to periodic pressure for 10,000 s, with a cycle time of 5 s. The inset graph further underscores the sensor’s rapid response to external pressure across different time scales, demonstrating excellent repeatability and high stability. Notably, the capacitance variation remained nearly constant after loading–unloading cycles, indicating the sensor’s high durability and accuracy. Thus, this stable sensor is poised to facilitate diverse applications in wearable electronics, human health monitoring, human–machine interfaces, intelligent robot, and beyond.

### 2.3. Filiform Cilia

Filiform cilia, with their slender morphology and high flexibility, offer extensive adaptability and dynamic sensing capabilities to sensors [36]. This type of cilia can bend without breaking when subjected to external forces, thereby enabling the effective perception of complex surface morphologies and dynamic changes. The application of filiform cilia in flexible sensors is mainly demonstrated in biomechanical sensing, surface topology detection, and other aspects. By simulating the delicate structure and movement mechanism of biological cilia, filiform cilia-based flexible sensors can precisely monitor and analyze the mechanical behaviors of cells, tissues, and even entire organisms [37].

**Figure 5:**
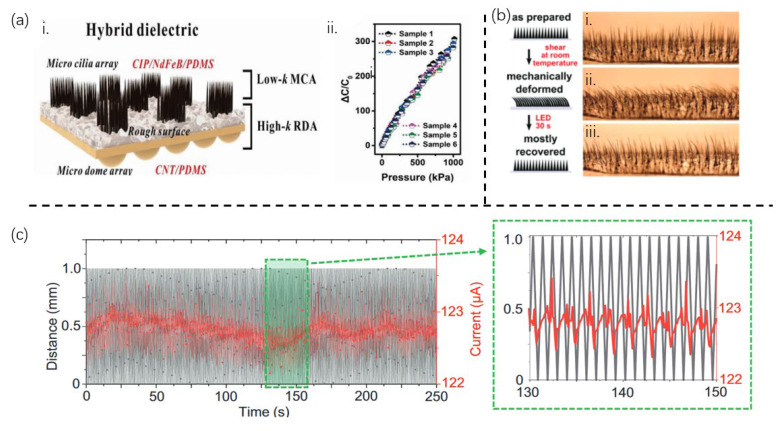
**Filiform cilia structures and their sensing performance.** (**a**) (**i**): A bio-inspired hybrid dielectric structure. (**ii**): Consistent high sensitivity and broad linear response across different samples, demonstrating the sensor’s reliability and scalability. (**b**) Mechanical deformation and shape recovery of the Diaplex magnetic cilia without a magnet. (**i**): The original, as-prepared shape. (**ii**): The mechanically deformed temporary shape obtained by shearing at room temperature without clamping or heating. (**iii**): The mostly recovered permanent shape obtained after turning on the LED for 4 min. (**c**) The cyclability test of a cilium bending toward another (d = 100 μm, l = 1 cm for both) in DI water over 250 cycles in 250 s, with current measured under a constant 2 V bias. Figure 5a, Figure 5b, and Figure 5c are from ref [21], ref [38], and ref [39], respectively.

Inspired by human skin, Ji et al. [21] developed a novel hybrid dielectric layer with gradient compressibility and dielectric properties as the active layer of a capacitive tactile sensor. This innovation effectively regulates capacitance changes, overcoming the limitations in sensitivity and linearity faced by traditional dielectric-based sensors due to difficulties in quantitatively controlling the interaction between mechanical and dielectric properties. The low-dielectric-constant layer is a CIP/NdFeB/PDMS film featuring a microcilia array (MCA) (Figure 5a-i), while the high-dielectric-constant layer is a CNT/PDMS film with a rough surface and micro-dome array (RDA). The pressure-induced series-parallel switching between the low-k and high-k films in the hybrid medium leads to a linear increase in the effective dielectric constant, allowing for the flexible adjustment of the initial/combined capacitance based on the optimized hybrid dielectric. This novel hybrid dielectric layer-based capacitive tactile sensor achieves a linearity of 0.99 at a line width of 1000 kPa (Figure 5a-ii) and a sensitivity of 0.314 kPa^−1^. The high sensitivity and ultrawide linear range are reproducible across different samples, indicating the excellent reproducibility of the proposed sensor device and method. Researchers also discovered that this hybrid dielectric layer is suitable for triboelectric tactile sensors. The adoption of the hybrid dielectric (the MCA with low κ, and the RDA with high κ) enhances triboelectric and electrostatic induction effects, achieving a linear relationship between output voltage and applied pressure. This results in triboelectric tactile sensors with an ultrawide linear range (1000 kPa) and significantly improved sensitivity.

Liu et al. [38] achieved advancements in the field of smart materials and programmable shape memory systems by ingeniously dispersing Fe magnetic particles into a thermoplastic polyurethane elastomer and shape memory polymer (SMP) matrix, creating a novel filamentous artificial magnetic cilia structure (Figure 5b). Under the influence of a magnetic field, the magnetic moments of the Fe particles interact, driving the entire cilia structure to self-assemble along the direction of the magnetic field. This self-assembly process not only enhances structural stability and uniformity but also enables the realization of complex shape transformations. Furthermore, remote stimulation through light and magnetic fields facilitates the reconfiguration of magnetic cilia, achieving intricate shape transformations and memory functions tailored to diverse application scenarios and requirements. Beyond its high controllability and reconfigurability, the combination of magnetic actuation and photothermal heating allows the magnetic cilia to switch between temporary and permanent shapes. The SMP cilia soften and deform into a temporary shape above the transition temperature but harden upon cooling, locking the shape at room temperature. By turning off light and the magnetic field, the temporary shape can be retained, while activating the light drive restores the permanent shape as needed. The permanent shape can also be reprogrammed by applying mechanical constraints and high-temperature annealing after cilia fabrication.

Glass et al. [39] provided an innovative and efficient solution for cellular mechanical sensing and sensor technology by demonstrating an artificial cilia array manufactured through 3D printing technology. These cilia not only exhibit high customizability but also serve as versatile tools for cellular mechanical sensing. This study employs extrusion-based 3D printing, utilizing graphene–polycaprolactone (PCL) composite ink as the printing material. This material combination endows the cilia array with high conductivity and excellent biocompatibility. With 3D printing, researchers can precisely control the size and shape of the cilia, notably achieving the high-aspect-ratio cilia arrays crucial for mimicking the intricate structures and functions of biological cilia. Combining finite element analysis and experimental results, researchers can accurately characterize the physical bending behavior of the cilia under linear pressure and external point forces. The cilia exhibit remarkable resilience, swiftly returning to their original position even after being bent 30°, a vital characteristic for achieving long-term stability and reliability in practical applications. The primary advantage of this mechanism lies in its scalability, customizability, and versatility, distinguishing it from other cell mechanical sensing cilia sensors based on magnetism or piezoelectricity. The bendable, conductive cilia can exist in both contact and separation states. By adjusting the material, size, and shape of the cilia, sensors suitable for various applications can be designed. The cilia sensor can detect various stimuli ranging from surface topology changes to airflow, water flow, and relative motion between two surfaces. A 250-cycle bending test (1 s per cycle) conducted in deionized water (Figure 5c) demonstrates that the cilia array maintains stable current readings after multiple cycles, proving its excellent durability and repeatability. It withstands high flow rates of up to 57 ms^−1^ in air and 240 mms^−1^ in water, consistently performing under numerous cyclic loads.

## 3. Fabrication Method of Artificial Cilia in E-Skin

Advancements in artificial cilia fabrication have yielded two main approaches: template-based and template-free. Template-based methods rely on acquiring appropriate templates, whereas template-free methods emphasize the scalability and reliability of production.

### 3.1. Template-Based Method

**Figure 6:**
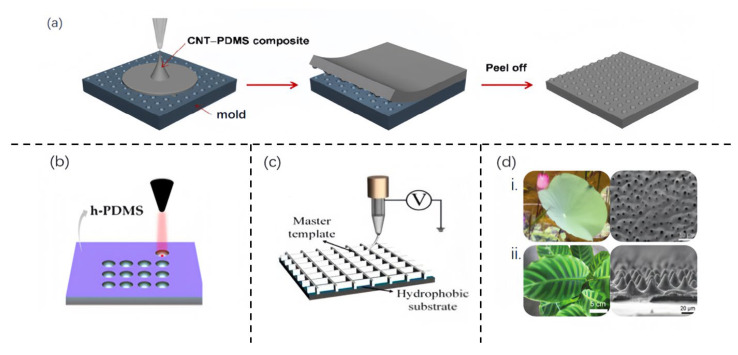
**Template-based fabrication methods.** (**a**) Conventional fabrication process of cilia. (**b**) Laser engraving on a h-PDMS mold for subtractive template manufacturing. (**c**) Electric field drive micro-scale 3D printing technology for fabricating a master template with a high aspect ratio, supporting additive template manufacturing. (**d**) (**i**): A photograph of a lotus leaf (**left**) for the natural production of a template and the top-view SEM image (**right**) after second molding. (**ii**): Photograph of a Calathea zebrine leaf (**left**) for the natural production of template and cross-sectional SEM images (**right**) after the second molding. Figure 6a, Figure 6b, Figure 6c, Figure 6d-i, and Figure 6d-ii are from ref [17], ref [41], ref [49], ref [34], and ref [53], respectively.

Over several years, various methodologies have been developed to create artificial cilia that mimic natural structures and functions. Among these, the template-based method has become a favored choice for scientists aiming to fabricate such structures [39]. A clear pattern emerges in their preparation [17]: a material is poured into a precisely crafted mold resembling the concave shape of cilia. Techniques such as heating, UV irradiation, or air drying are then used to solidify the material, retaining the desired form. After solidification, the mold is removed, leaving the newly formed cilia (Figure 6a). This conventional pouring method is straightforward. However, to create cilia that better meet specific expectations, research teams have refined the mold production process, categorizing their methodologies into three approaches: subtractive fabricating [31,32,33,34,35,36,37,38,39,40,41,42,43,44,45,46,47], additive fabricating [48,49,50], and natural making [34,51,52,53]. Each builds on the foundational pouring method, introducing innovations to achieve higher precision and fidelity in replicating natural cilia structures.

#### 3.1.1. Subtractive Manufacturing

Laser engraving, as a common subtractive manufacturing process, necessitates the initial design of an engraving pattern. For instance, Andreia dos Santos [41] and her colleagues designed patterns consisting of circles with diameters of 100 μm or 200 μm, and spacings of 150 μm or 200 μm. This was repeated over a 2 cm × 2 cm area. Once the pattern was ready, a rigid PDMS mold was created by mixing the PDMS curing agent and elastomer at a ratio of 1:5 w/w, degassing the mixture in a vacuum for 30 min, pouring it into a Petri dish, and then heating and curing it for an hour. Subsequently, the mold underwent microstructuring using a laser engraving machine (Figure 6b).

The photolithography is another normal process of subtractive manufacturing, and it can be divided into two stages of imaging and etching. During the imaging stage, a photoresist layer is uniformly coated onto the substrate and is subsequently covered with a masking material. The photoresist layer is then exposed to ultraviolet radiation, ensuring that the photoresist pattern matches the mask design. After the mask removal, the substrate undergoes etching in a specific solution, followed by the removal of the photoresist layer. For instance [45], Yuan and his team initiated the process by cleansing a silicon wafer. We applied the photoresist mask and the desired pattern to the wafer using a mask aligner. The wafer was then baked at a high temperature and we etched the exposed silica, resulting in the formation of a silica grid on its surface. The photoresist mask was then stripped. Lastly, the silicon wafer underwent treatment with a potassium hydroxide solution to create cavities within the cilia array.

The subtractive method is favored for large-scale cilia production, but it generates smoke and dust, posing health risks, especially during laser processing [46]. Photolithography is limited by mask patterns, reducing manufacturing freedom for complex shapes. Additionally, subtractive processing involves multiple steps, which are time-consuming and generate significant waste.

#### 3.1.2. Additive Manufacturing

The emergence of 3D-printed micro-templates offers a solution to the drawbacks of subtractive manufacturing, based on the principle of rapid in situ forming, known as “additive technology” [48]. Peng et al. introduced a groundbreaking process for the fabrication of wax-based material microstructures, leveraging electric field-driven microscale 3D printing [49], as shown in Figure 6c. This technique was successfully applied to the production of microfluidic chips. The authors proposed a cost-effective and easily fabricable polymer micro-mold characterized by a high aspect ratio (AR), facilitated by electric field drive microscale 3D printing. This entire production workflow dispenses with the need for expensive equipment, enabling the rapid and mass-scale production of large-area, ultra-fine, and high-AR molds at ambient temperature.

However, it is imperative to highlight several points, based on domestic and international research, and their applications [49]. Firstly, despite the rapid development of additive manufacturing technology, its level of technological maturity still lags behind that of traditional processing methods. Consequently, there remains a certain gap in terms of how widely it can be applied. Secondly, with regard to printing materials, while additive manufacturing itself places limited restrictions on materials, the need for printing molds that require certain robustness and durability poses challenges. Therefore, materials that are too soft, such as hydrogels, are not suitable for preparing molds through additive manufacturing; similarly, materials that are too bulky, like aluminum, are also inappropriate. Thus, additive manufacturing is also constrained by the common limitation of templating methods, with restricted material selection. Lastly, from a cost perspective, existing 3D printers remain generally expensive, rendering them unsuitable for large-scale production endeavors. Despite these challenges, the potential of 3D printing technology continues to be explored and refined, paving the way for future advancements and innovations in various industries.

#### 3.1.3. Natural Making

While the two template methods introduced earlier for the fabrication of cilia structures do indeed possess certain defects, varying in severity, they both, on balance, ensure that the resultant microstructures exhibit commendable homogeneity. This homogeneity is a crucial aspect, as it guarantees consistency in the performance and behavior of the cilia structures across different applications. In recent years, researchers have explored a suite of natural production methods through which the template can create cilia arrays.

Focusing attention on plants with complex patterns on their surfaces, the lotus leaf and Calathea zebrine leaf stand out as a particularly popular templates with which to fabricate the microstructure of the flexible sensors. This is primarily attributed to its surface, which is adorned with papillae measuring approximately 10 microns in size. These natural, columnar structures possess a low density and a high aspect ratio, making them highly suitable for a range of purposes. C. F. Guo and his colleagues showcased the effectiveness of a biomimetic micropatterned m-PDMS [34], which was replicated from lotus leaves (Figure 6d-i). When coated with ultrafine silver nanowires, serving as the bottom electrode, this material enhances the performance of capacitive sensing devices. The resulting device exhibits a remarkable sensitivity of around 1.2 kPa^−1^, an ultra-low detection limit of less than 0.8 Pa, and a swift response time of 36 milliseconds. Furthermore, this flexible tactile sensor demonstrates exceptional durability, enduring at least 100,000 cycles without any signs of fatigue. Qiu et al. have taken an innovative approach by using the Calathea zebrine leaf as a template (Figure 6d-ii) to fabricate a microstructured ionic gel (MIG) film [53]. This film is ingeniously utilized as the dielectric layer in a flexible ionic skin. Through a precise templating and replication process, the surface of the MIG film is adorned with highly uniform microcones. These structures not only replace the traditional, complex micro-pyramid designs but also offer unprecedented performance advantages. This flexible MIG skin exhibits a remarkable performance in response time, detection limit, and sensitivity. With a swift response time of 29 milliseconds, an ultra-low detection limit of 0.1 Pa, and an exceptionally high sensitivity exceeding 54.3 kPa^−1^ within the extremely low-pressure range below 0.5 kPa, it significantly outperforms current technologies. Notably, its robustness stands out, maintaining stability through thousands of compression cycles. This pioneering advancement is attributed to a unique ion-electron capacitive interface that enables precise pressure sensing by modulating the double-layer electrical (EDL) interface area upon compression.

The adoption of natural plant templates for cilia fabrication boasts simplicity and efficiency, yet the resultant structures display irregular morphologies, sizes, and spacings, factors which undermine the device’s performance consistency. Moreover, scalable, customized production poses significant hurdles.

### 3.2. Template-Free Method

Template-free preparation is an approach that involves the direct formation of the desired structure via chemical self-assembly, magnetic self-assembly, or other physical mechanisms, all without relying on a pre-established template [54,55,56,57]. This innovative approach offers enormous advantages in terms of simplifying the manufacturing process, enhancing overall production efficiency, and saving substantial costs.

#### 3.2.1. Chemical Self-Assembly

**Figure 7:**
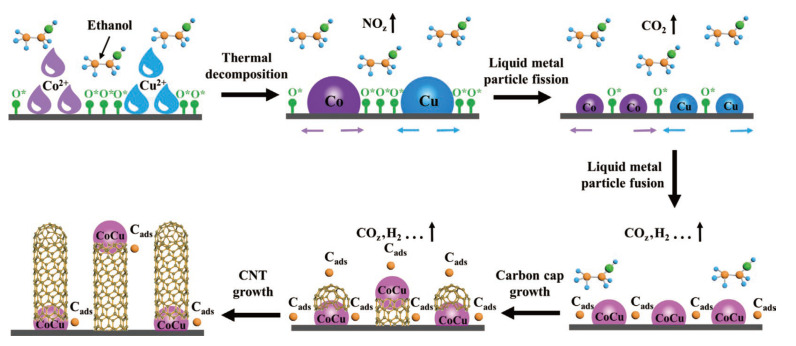
**Chemical self-assembly**: sequential schematic illustrating the chemical transformations during CNT growth. O*, NO_z_, and CO_z_ represent the residual oxygen, the mixture of NO and NO_2_, and the mixture of CO and CO_2_, respectively. This picture is from ref [54].

In a pioneering study conducted by Wang et al. (Figure 7) [54], a novel ultrafast carbon thermal shock (CTS) method was developed in order to synthesize carbon nanotubes (CNTs). This method was executed with a simplistic, homemade device that harnessed the power of carbon-based Joule heating. The researchers utilized carbonized silk fabrics (CSFs), impregnated with transition metal salts, that were dissolved in an ethanol solution as the foundational substrates. Through adjusting the two primary parameters, namely voltage and pulse width, and subjecting the samples to an impulse voltage of 40 V for 50 milliseconds, the authors successfully grew CNTs adorned with bimetallic alloy catalyst nanoparticles. These boasted diameters of approximately 9 nm. The resultant material exhibited a fluffy structure, bearing a striking resemblance to the intricate spider cup hair. This innovative CNT material was then transformed into a highly sensitive airflow sensor and an efficient air filter, underscoring its broad potential for diverse applications. In another distinct yet equally groundbreaking study, Zhang et al. [57] devised a multifunctional electrospinning microcone array (EMPA), integrating it with an ultra-thin, lightweight, and breathable architecture. This was accomplished through a self-assembly technique leveraging the charged jetting of wet heterostructures. EMPA’s standout feature lies in its piezocapacitive triboelectric hybrid sensor, which demonstrates a remarkable ability to detect extremely subtle fingertip pulses. This ability is crucial for health diagnostics, allowing the sensor to perceive pulses across a wide range of frequencies with exceptional sensitivity (19 kPa^−1^), an exceptionally low detection threshold (0.05 Pa), and an extremely swift response time (0.8 ms). These attributes position EMPA as a highly promising candidate for applications in E-skin technology.

It is important to note that a large number of cilia produced by chemical self-assembly are generally disordered, i.e., their morphology is difficult to effectively control. At the same time, this method requires the use of complex and expensive equipment, and it can lead to a potential pollution issue, which needs to be carefully considered and managed in the future.

#### 3.2.2. Magnetic Self-Assembly

**Figure 8:**
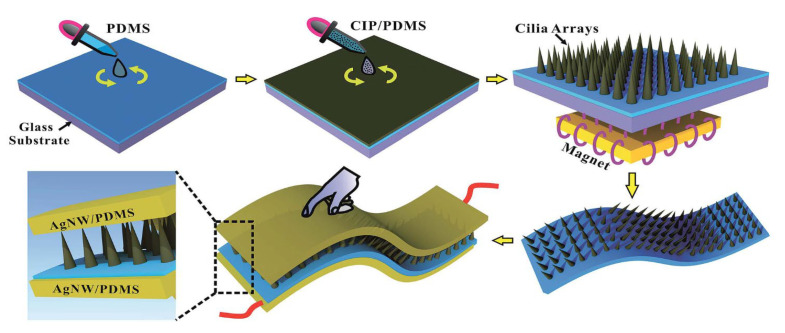
**Magnetic self-assembly.** The schematic of the fabrication process of the capacitive pressure sensor, using the MCA as the dielectric layer. This picture is from ref [35].

In recent years, the magnetic-assisted construction of artificial cilia has emerged as a promising method with which to complement chemical self-assembly [58,59,60,61,62,63,64], drawing significant interest from researchers in many fields because it displays the characteristic whereby magnetic particles are rapidly polarized to order the arrangement under the action of a magnetic field, forming a microstructure of cilia with a large aspect ratio at the interface. A pioneering example of this technique was demonstrated by Zhou and colleagues [35], who successfully fabricated a flexible capacitive pressure sensor that utilized a microcilia array as its dielectric layer. This innovative sensor was created using the magnetic field generated by a portable permanent magnet (Figure 8), highlighting the simplicity and cost-effectiveness of the method. Through this approach, parameters such as the type of magnetic particles, the PDMS/CIP mass ratio, and the film thickness could be easily manipulated and optimized, allowing for the creation of MCA (microcilia array) dielectric layers with precise and controlled shapes. The resultant MCAs exhibited exceptional flexibility and recoverability under cyclic external high-pressure loading and unloading conditions. Its detection range spanned up to 200 kPa, with a sensitivity of 0.02 kPa^−1^ within the 50 to 200 kPa range, and a remarkable detection limit of 2 Pa. During the test of pressure cycle, the MCAs displayed outstanding structural robustness and stability.

Asghar et al. extended this methodology by mixing silver-clad nickel particles (Ag@Ni) with PDMS and placing the mixture in a magnetic field, thereby obtaining cilia-like microstructures [9]. Compared to previous microcilia structure preparation processes, the magnetic self-assembly method offered a streamlined and more environmentally friendly alternative. However, despite its advantages, this technique was not without its limitations. The simple static magnetic field used in these studies lacked the regulation and programmability necessary for optimizing the growth of microstructures. Consequently, the magnetic-assisted method was unable to maximize the performance of the sensors to their fullest potential. To solve this challenge, Xu et al. [8] introduced an innovative ultrafast method for fabricating ordered cilia arrays, leveraging the unique effects induced by microcavities. By skillfully integrating static and dynamic magnetic fields, they initially formed delicate cilia in the outer area of the microcavity. These cilia then converged above the microcavity, ultimately coalescing into a fully formed cilia structure, and so the ordered cilia array could be formed within 30 s.

The height–diameter ratio of the cilia structure can be adjusted within a wide range by magnetic self-assembly, providing a versatile platform for the large-scale production of artificial cilia tailored to various applications.

#### 3.2.3. Three-Dimensional Printing

Despite the magnetic self-assembly method being recognized as a highly ideal preparation technique, a group of pioneering scientists have elected to explore the utilization of 3D printing technology [65,66,67,68,69,70] in order to fulfill their unique customization requirements for cilia. This decision stems from the unparalleled capabilities of 3D printing, which allow for precise material deposition at the micron scale. As a result, ciliary structures can be printed with exceptional accuracy and detail [70]. Leveraging advanced 3D modeling software and sophisticated algorithms, researchers are granted unparalleled freedom to design the various parameters of cilia, including their shape, size, orientation, and distribution density.

**Figure 9:**
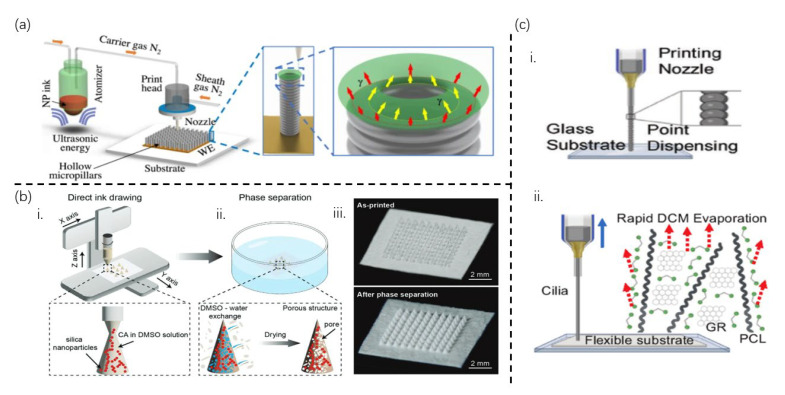
**Three-dimensional printing.** (**a**): The AJ 3D printer uses an ultrasonic atomizer to convert metal nanoparticle ink into an aerosol. The aerosol is transported to the nozzle by N_2_ carrier gas and focused on the substrate by a N_2_ sheath gas at a 10 μm scale. Hollow micropillars are formed by the layer-by-layer printing of concentric toroid-shaped ink rings. Surface tension (red and yellow arrows) aids structure buildup. Each layer solidifies upon solvent evaporation from platen heat, providing a base for the next layer. (**b**) (**i**,**ii**): The fabrication of porous microconical cilia. Schematic of the direct ink drawing process for nanocomposite inks, followed by coagulation in a deionized water bath to produce microconical cilia. (**iii**): An optical image showing as-printed microconical cilia in a 10 × 10 square array on a filter paper substrate and the corresponding microconical artificial cilia patch after phase inversion. (**c**) (**i**): This point-dispensing methodology allows for aspect ratios up to 15:1 (**ii**): Rapid DCM evaporation from a graphene/PCL nanocomposite ink enables the formation of high-aspect-ratio cilia. Printing is performed on a flexible substrate. Figure 9a, Figure 9b, Figure 9c-i, and Figure 9c-ii are from ref [66], ref [65], ref [67], and ref [39], respectively.

Ali and colleagues successfully fabricated a hollow micro-columnar artificial cilia array using aerosol jet (AJ) nanoparticle 3D printing technology [66], as shown in Figure 9a. Meanwhile, Pang et al. proposed a cost-effective and convenient manufacturing method for porous microconical artificial cilia arrays [65]. By developing nanocomposite inks in the form of yield stress fluids, which are formulated by incorporating silica nanoparticles into a cellulose acetate solution, the 3D printing process has been streamlined (Figure 9b). This solution was then subjected to a controlled drawing process where it was squeezed and lifted to form individual conical cilia, ultimately resulting in customized artificial cilia patches. Subsequently, interconnected porous microstructures were created through phase separation in deionized water. The additive manufacturing method established in this study simplifies the fabrication of porous artificial cilia patches, enabling less invasive collection of interstitial fluid (ISF) as flexible patches for sampling from non-flat skin sites. This microconical cilia processing technology significantly enhances the efficiency of manufacturing compared to traditional additive manufacturing methods that involve the printing of stacks layer by layer. Moreover, it transcends the limitations of templates, allowing for the customization of cilia arrays of any shape and size according to predetermined programs.

In other significant studies, Shar et al. reported a low-cost, room-temperature method for the preparation of one-component, conductive, and flexible carbon nanotube (CNT)–silicone 3D printing inks, as shown in Figure 9c-i [67]. The use of butyl acetate (BA) as a common solvent for CNT and one-component room-temperature vulcanized (RTV) silicones offer a low-toxicity, biologically derived approach to CNT dispersion. Additionally, the one-component humidity curing mechanism, facilitated by the chemical reaction between atmospheric humidity and silicones, makes the ink more manageable than the previous two-part PDMS-based conductive ink. The specific printing process involves dispensing the CNT–silicon ink for approximately 0.5 s through the 3D printer, followed by a pause of about 0.3 s. The printer then moves up to a set distance of approximately 300 μm and continues the dispensing cycle. Through numerous cycles, multiple uniform spheres are formed, which merge to create a tower with a high aspect ratio. A remarkable length-to-diameter ratio of 15:1 was achieved. However, it is important to note that since cilia are composed of dot inks, their uniformity and stability may be somewhat limited. Consequently, there is a need to improve the aspect ratio and resolution of cilia to ensure optimal performance. Glass et al. proposed an innovative polycaprolactone (PCL)/graphene cilia sensing array that seamlessly integrated the controllable and straightforward processing of 3D printing with contact resistance-based sensing (Figure 9c-ii) [39]. This approach enables the creation of highly customizable artificial cilia with filiform structure, boasting a broad spectrum of lengths ranging from 1 to 20 mm and diameters spanning from 100 μm to 1 mm. Such versatility allows for precise control and adjustable high aspect ratios, reaching up to approximately 200. By printing these cilia directly onto a flexible adhesive substrate featuring a rubber layer, with the cilia attached to a silver electrode, meticulous control over the sensor’s geometry is attainable. This ensures the accommodation of various target sizes and contours, thereby enhancing the adaptability and practicality of the sensing array.

## 4. Applications

As discussed previously, cilia-inspired bionic e-skin has opened up a wide range of potential applications. This section focuses on how it can be used in healthcare and motion detection, human–machine interaction, and the design of dexterous hands, and can also be used in near-field communication and perceptual decoupling [70,71,72,73,74,75,76,77,78,79,80,81,82,83,84].

### 4.1. Healthcare and Motion Detection

**Figure 10:**
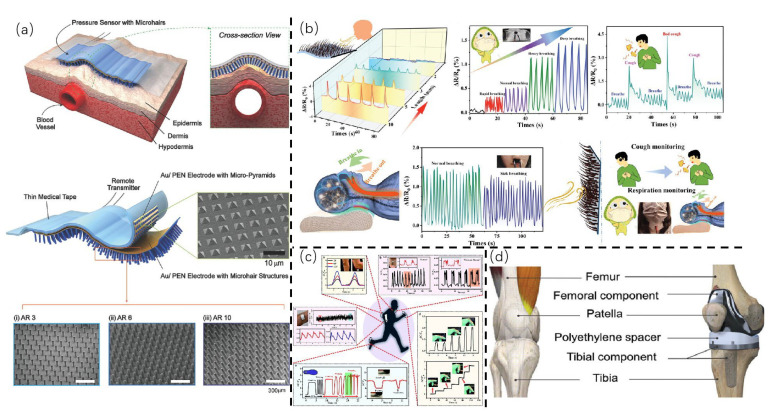
**Applications in healthcare and motion detection.** (**a**) A schematic illustration of the method used to detect pulse on a human’s neck with our microhair sensor and the pyramid-shaped PDMS dielectric layer, which was placed between the two Au electrodes on PEN plastic substrates. The pressure sensor was subsequently added on a layer of microhair-structured biocompatible polymers (PDMS) to enhance skin conformity. SEM images of dense microhair arrays with 30 μm radius and AR values of (**i**) 3, (**ii**) 6, and (**iii**), 10. (**b**) Long cilia P-silk/RG E-skin monitors human respiration, including breathing and coughing, and performs noncontact sensing for disease diagnosis and treatment. (**c**) Real-time signal of the sensor based on the MCA, which is attached to various parts of the human body to monitor human’s common movements. This includes the real-time monitoring of flexible elbow bending and relaxation with different bending angles; the real-time monitoring of voice vibration during the pronunciations of the ‘sensor’ and ‘pressure sensor’; the real-time wrist pulse monitoring test of an adult volunteer for 1 min; and the real-time monitoring measurement of periodic finger bending, relaxation, and long-time bending with different bending angles and real-time pressure monitoring of the hind sole with different weights (55 kg and 75 kg) in different motion states (standing, walking and jumping). (**d**) A schematic showing the replacement of the femur or tibia by the metal prosthesis during TKA surgery. Figure 10a, Figure 10b, Figure 10c, and Figure 10d are from ref [86], ref [87]. Ref [35], and ref [88], respectively.

The maintenance of good health remains a fundamental concern for humanity, and the use of cilia-inspired bionic tactile E-skin can provide key physiological data for this aim. Common physiological signals are weak signals consisting of the pulse and breathing. As shown in Figure 10a, Bao et al. reported a flexible pressure sensor based on a ciliated structure [85]. The sensor exhibits super-compliance and an approximately 12-fold increase in signal-to-noise ratio for the generated capacitive signals, enabling the measurement of weak pulsations from the internal jugular vein. In the test, the fabricated devices showed excellent stability of sensitivity (0.56 kPa^−1^) after a prolonged cycling test, and so it still accurately detects the capacitance change in arterial pressure. It is noteworthy that ciliated microstructures are highly effective in detecting airflow. Consequently, the application of cilia-inspired bionic tactile e-skin to flow sensing has yielded excellent results. Yu et al. reported a polypyrrole silk/glycerol plasticized silk fibroin (P-silk/RG) e-skin fabricated by a simple 3D biomimetic structural strategy [86]. Benefitting from the editability (length, position) of this structure, P-silk/RG has signal selectivity. Long-cilia P-silk/RG demonstrates high sensitivity (responding to weak-signal airflow), while the short-cilia P-silk/RG exhibits a wide pressure detection range (0.5–200 g) and high cycle stability (8000 times). The P-silk/RG with different forms can be used for the detection of different types of physiological signals. For instance, in the field of flow sensing, the P-silk/RG e-skin with long cilia is capable of distinguishing between normal breathing, aggravated breathing, deep breathing, and coughing (Figure 10b), which makes it a potentially valuable tool for health monitoring and early warning.

In terms of body movements, Zhou et al. reported a flexible capacitive pressure sensor using a hair-like microcilia array (MCA) as the dielectric layer through a facile and cost-effective methodology. The sensor displays high sensitivity (0.28 kPa^−1^), a broad sensing range (0–200 kPa), a good detection limit (2 Pa), and excellent structural robustness and stability [35]. From Figure 10c, it can be found the sensor is capable of detecting a multitude of body movements, including finger movements, elbow bending, foot lifting and lowering, and throat vibration and pulse beating. Similarly, the team of Huang exploited the strain effect of the sensing layer caused by the staggered microcilia to fabricate a flexible sensor with increased sensitivity (5.22–70 MPa^−1^) over an ultrawide pressure range (45 Pa–4.1 MPa), and it also displayed a high-pressure resolution (5 Pa), a fast response/recovery time (30/45 ms), and a robust response under a high-pressure loading of 3.5 MPa for more than 5000 cycles [87]. The use of the device was demonstrated in TKA surgery (Figure 10d), helping clinicians to restore neutral mechanical axes, monitor knee flexion angles, and extract gait characteristics.

### 4.2. Human Machine Interaction and Dexterous Hand

Recently, there has been flourishing application of neural networks and deep learning in flexible e-skin [88,89], dramatically increasing the study of human–machine interactions and dexterous hands. Therefore, cilia-inspired bionic e-skin with precise and advanced sensing has huge application potential.

**Figure 11:**
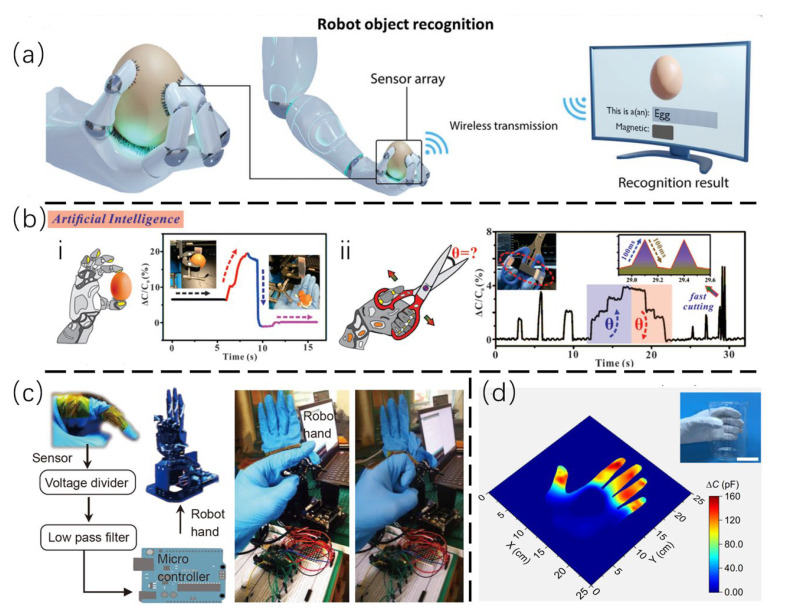
**Applications in human–machine interaction and dexterous hands.** (**a**) Schematic diagram of object recognition with sensors. After the robotic arm with the sensor grabs an object, the recognition results, including whether the object is magnetic, are displayed on a screen (**b**). (**i**): Optical images and real-time capacitance variation from the sensor mounted on the clamp during the process of grasping and crushing an eggshell. (**ii**): Real-time recognition of the scissor status with the attached shear sensor in terms of cutting speed and angle monitoring. The inset demonstrates the sensor’s rapid response to shear forces within ~100 ms (**c**). The HMI application of the sensor. (**d**) All-textile smart glove for pressure mapping and pulse wave detection. Capacitance mapping of the smart glove worn on a healthy subject holding a beaker. Scale bar: 5 cm. Figure 11a, Figure 11b, Figure 11c, and Figure 11d are from ref. [30], ref. [58], ref. [59], and ref. [6], respectively.

In this respect, Chen et al. reported a flexible tactile sensor with an innovative design of magnetic cilia array, which provides excellent and stable sensitivity for human–machine interactions and dexterous hands [30]. The sensor has a resolution of 0.2 mN with a working range of 0–19.5 mN, and it is able to distinguish the direction of external force that can be applied in object recognition with a success accuracy of 97%. In addition to the shape of objects, the sensor can identify whether there is magnetism inside objects (Figure 11a), making it a highly valuable tool in the development of intelligent robots and in the field of modern medicine. Similarly, Zhou et al. fabricated a magnetic field-assisted in situ formation spine array as the dielectric layer of the capacitive sensor. This capacitive sensor possesses a sensitive pressure monitoring function, which allows it to respond to normal forward pressure and small shear forces [58]. In the demonstration (Figure 11b), the clamp was used to mimic the mechanical hand that can hold the eggshell stably, and it is adhered to the handles of scissors for cutting recognition.

Furthermore, Wang et al. developed a sensor based on nickel fabric electrodes, incorporating magnetic field-driven porous elastomers with micropillar arrays (MPAs) as sensitive elements [59]. As shown in Figure 11c, the distinctive configuration of the MPAs and the refined texture of the electrodes enable the sensor to demonstrate an exceptional degree of sensitivity, reaching 10,268 kPa^−1^, and the capability of discerning minute pressure fluctuations as low as 0.25 Pa. Moreover, this sensor system is combines with wireless charging via the use of a flexible supercapacitor, which contributes to its exceptional overall performance. In the application, the robot hand was successfully operated via the establishment of a Bluetooth connection between the sensor and the robot hand, resulting in synchronized movement between the two. It demonstrates considerable potential for the realization of human–machine synchronization. In addition, Guo et al. constructed a simple skin electrode mechanical sensing structure (SEMS) [6]. The experimental results demonstrate that gloves equipped with the SEMS structure are capable of accurately mapping the pressure distribution and discerning changes in object hardness with precision (Figure 11d). It is anticipated that with the aid of sophisticated data analysis and machine learning techniques, these gloves will evolve into a groundbreaking tool that can accurately identify the hardness and shape of objects, while also facilitating the recovery of operational tasks and object recognition abilities in patients with impaired touch. This could be regarded as an exemplary platform in the domain of tactile rehabilitation. Furthermore, the wearable haptic device presents a multitude of possibilities for health monitoring, including the observation of blood flow and pulse, as well as the tracking of motion and the facilitation of seamless human–machine interaction.

### 4.3. Near-Field Communication and Perceptual Decoupling

**Figure 12:**
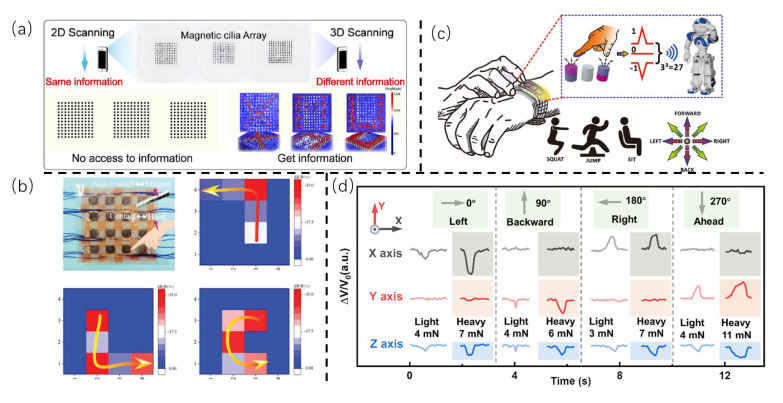
**Applications in near-field communication and perceptual decoupling.** (**a**) Microcavity-induced magnetic cilia array (CI-MCA) 3D code and information security. (**b**) Non-contact sensing and tactile-perception behavior of L-MPF electronics. (**c**) Proposed high-capacity transmitter for outputting multi-bit coded control instructions based on the sensor. (**d**) Tactile monitoring: different strengths and directions of shear force imposed by finger. Figure 12a, Figure 12b, Figure 12c, and Figure 12d are from ref. [8], ref. [63], ref. [92], and ref. [61], respectively.

The term “near-field communication” is used to describe the transmission of data or interaction between devices through the use of specific wireless communication technologies over relatively short distances. By employing variations in the electric [90,91] and magnetic [63,92,93] fields of the e-skin, researchers have begun to explore the integration of near-field communication technology into bionic E-skin. Owing to the magnetic-assisted fabrication of cilia-inspired bionic tactile e-skin, the design of magnetic cilia array has great potential applications in near-field communication through the use of encryption technology and contactless signaling mechanisms. As shown in Figure 12a, depicting encryption, the cilia array is specifically designed to possess three-dimensional properties throughout the magnetic field [8]. The ordered cilia array can be used as a fast, secure information carrier. These arrays are designed to create 3D codes, which cannot be scanned with regular 2D technology, addressing security concerns related to the use and duplication of traditional 2D codes.

It is noteworthy that Gong’s team developed an ultra-sensitive multi-function sensor that ingeniously integrates tactile and magnetic sensing technologies into a single device [63], designated as L-MPF. The sensor comprises two intertwined hair-like magnetizing elements that are self-assembled under a magnetic field and under pressure. The L-MPF sensor has excellent magnetic field detection capabilities and can also measure different types of loads, such as pressure, shear force, and magnetic fields. The sensitivity coefficient can reach 1965, 1.6, and 2.4 T^−1^ under 50–60% compressive strain, 0–3.4% shear strain, and 21–170 mT magnetic field. As shown in Figure 12b, when used as a 4 × 4 array, the L-MPF sensor can recognize contactless gestures, opening up new possibilities for advanced human–machine interaction and the development of bionic E-skin.

Furthermore, Zhou et al. reported a new capacitive sensor with a flexible micromagnet array (t-FMA) that drives the interaction [92]. This sensor can bend in two directions and allows for the real-time measurement of both the strength and direction of a magnetic field, without interference from capacitive signals. The sensor displays a sensitivity of over 1.3 T^−1^ and the ability to detect weak magnetic fields as low as 1 mT with excellent durability (over 10,000 cycles). As shown in Figure 12c, the sensor is integrated into systems like contactless Morse code input and Braille communication interfaces, showing its potential in real-world applications. In the field of perceptual decoupling, this sensor can also accurately measure the direction and strength of the magnetic field, making it useful for applications like encrypted ID recognition and multi-command control.

Similarly, as shown in Figure 12d depicting perceptual decoupling, Zhang et al. reported a shear force sensor constructed by a magnetically induced assembled Ni/PDMS composite membrane, which is magnetized and integrated with a three-axis Hall sensor, facilitating its ability to simultaneously monitor both shear force magnitude (0.7–87 mN) and direction (0–360°) [61]. The cilia-inspired shear force magnetic sensor (CISFMS) exhibits admirable attributes, including exceptional flexibility, high sensitivity (0.76 mN^–1^), an exceedingly low detection limit (1° and 0.7 mN), and remarkable durability (over 10,000 bending cycles). This work demonstrates the capacity of the CISFMS in detecting tactile properties, flow sensing, and direction.

## 5. Summary and Outlook

Recent advancements in bionic tactile E-skin have been profoundly influenced by the cilia structures found in nature, driving significant progress in refining fabrication processes, enhancing materials, and expanding application areas [93,94,95,96,97]. This review provides a comprehensive analysis of the diverse morphologies, fabrication techniques, and applications of cilia-inspired bionic tactile E-skin, detailing key microstructures, including columnar, conical, and filamentous cilia. While microstructure design can substantially improve device performance, inherent trade-offs make it challenging to simultaneously optimize sensitivity, response time, operating range, and stability. Conical-structured sensors exhibit high sensitivity and linearity in low-pressure ranges but suffer reduced performance at higher pressures and a limited measurement range. Columnar structures generally ensure good linearity due to their uniform shape but often do so at the cost of sensitivity. Filamentous cilia offer superior properties due to their high aspect ratio, yet current designs typically feature arrays of independent cilia rather than branching structures that are similar to natural feathered cilia. This gap presents a potential avenue for future research.

Through specific examples, we have elucidated and synthesized production techniques for microcilia, assessing the strengths and limitations of various approaches to offer a foundation for further research. Although current research primarily emphasizes high precision, balancing precision with efficiency remains a challenge. Additionally, existing microfabrication techniques are either too complex or costly to enable scalable production, underscoring the need for diversified, scalable methods. Therefore, the large-scale, integrated, high-speed, and low-cost fabrication of microcilia remains a significant challenge in the field of scientific research. Furthermore, material selection for manufacturing also merits further exploration; replicating the properties of natural materials often relies on their intrinsic characteristics, which can hinder additional design flexibility. While the use of biomaterials has been explored, their inherent instability and material-specific limitations restrict their applicability in certain scenarios. Future research must address the challenge of identifying suitable, stable materials that can withstand diverse operational demands.

In conclusion, advancements in cilia-inspired bionic tactile E-skin are expected to broaden its application scope into more nuanced and sensitive areas, including aerospace, environmental monitoring, and daily-life applications. However, natural organisms are highly complex systems, and despite significant strides in flexible E-skin technology, a substantial gap remains between synthetic skins and biological skin. Therefore, solving the challenge of the fabrication, realizing the industrialization of cilia-inspired bionic tactile E-skin, and exploring whether its performance might even surpass that of its biological counterpart represent key directions for future exploration and potential breakthrough.

## Data Availability

Not applicable.
